# Autophagic flux, a possible mechanism for delayed gentamicin-induced ototoxicity

**DOI:** 10.1038/srep41356

**Published:** 2017-02-01

**Authors:** Yeon Ju Kim, Chunjie Tian, Jangho Kim, Beomyong Shin, Oak-Sung Choo, You-Sun Kim, Yun-Hoon Choung

**Affiliations:** 1 Department of Otolaryngology, Ajou University School of Medicine, San 5 Woncheon-dong, Yeongtong-gu, Suwon 16499, Republic of Korea; 2 Department of Otolaryngology, Dali Bai Autonomous Prefecture People’s Hospital, Renminnan road 35, Dali, Yunnan 671000, China; 3 Department of Rural and Biosystems Engineering, Chonnam National University, Gwangju 61186, Republic of Korea; 4 Department of Biomedical Sciences, BK21 Plus Research Center for Biomedical Sciences, Ajou University Graduate School of Medicine, San 5 Woncheon-dong, Yeongtong-gu, Suwon 16499, Republic of Korea; 5 Department of Medical Sciences, Ajou University Graduate School of Medicine, San 5 Woncheon-dong, Yeongtong-gu, Suwon 16499, Republic of Korea

## Abstract

Aminoglycoside antibiotics including gentamicin (GM) induce delayed ototoxic effects such as hearing loss after long-term use, unlike the early-onset ototoxicity caused by cisplatin. The purpose of the study was to identify the mechanism of the delayed GM-induced ototoxicity by exploring the role of autophagy *in vitro* and *in vivo*. Treating HEI-OC1 auditory cells with GM led to a time-dependent increase of the autophagosome marker LC3-II, which was accompanied by cell death. In contrast, cisplatin and penicillin caused a rapid increase and had no effect on LC3-II levels, respectively. LC3-II-expressing autophagosomes co-localized with the labeled GM. GM-treated autophagosomes expressed reduced levels of Rab7, which is necessary for the fusion of autophagosomes with lysosomes. When the autophagic flux enhancer rapamycin was applied to GM-treated cells, Rab7 and the lysosomal enzyme cathepsin D were upregulated, and increased cell survival was observed. In animal studies, the intraperitoneal injection of GM worsened hearing thresholds and induced the accumulation of LC3 in the organ of Corti. This hearing impairment was attenuated by rapamycin. These findings suggest that the delayed onset-ototoxicity of GM may be closely related to the accumulation of autophagosomes via impaired autophagy. This GM-induced auditory cell death could be inhibited by enhancing autophagic flux.

Aminoglycoside antibiotics have been used clinically to treat various infectious diseases, including those caused by gram-negative bacteria. However, the clinical use of aminoglycoside antibiotics is limited because of serious adverse effects and particularly ototoxicity, which can result in hearing loss, tinnitus, and vestibular disorders^1^. Among the available aminoglycosides, gentamicin (GM) is an efficient drug due to its low bacterial resistance and reasonable cost^2^. The prevalence of ototoxicity in patients receiving GM ranges from 2 to 25%^3^. GM-induced ototoxicity occurs sporadically in a dose-dependent manner, and once hearing is damaged the likelihood of recovery is very low. Interestingly, the onset of the symptoms is typically delayed until days or months after GM administration^4^. In contrast, cisplatin exhibits acute ototoxic effects. The intracellular damage caused by aminoglycoside and cisplatin seems to share a final common pathway; however, the upstream cell death events depend on the individual ototoxic insult^5^.

The most common reason for GM-induced hearing impairment is the loss or dysfunction of hair cells, which are highly specialized sensory receptors that transform a mechanical stimulus into nerve impulses for hearing^6^. Histological and ultra-structural studies have shown that GM is preferentially driven into hair cells by endocytosis or through mechanoelectrical transduction channels^7^,^8^. Recent studies have suggested that the dysregulation of cytoplasmic and mitochondrial protein synthesis plays an important role in aminoglycoside ototoxicity^9^. Impaired protein synthesis in hair cells, resulting from aminoglycoside treatment-induced defective mitochondrial function, activated the c-Jun N-terminal kinase (JNK) pathway^9^ and induced reactive oxygen species (ROS) formation^10^,^11^. However, the ototoxicity of aminoglycosides, including GM, remains poorly understood, and particularly the reasons for the delayed onset of GM-induced ototoxicity.

Autophagy is a major catabolic process that plays an important role in balancing protein synthesis, protein degradation, and the recycling of cellular components via self-digestion^12^. Autophagy functions mainly as a cell survival pathway by degrading long-lived and misfolded proteins and damaged organelles to generate the amino acids for energy utilization, particularly under stress conditions such as starvation and hypoxia^13^. Nevertheless, excessive or impaired autophagic flux can contribute to cell death via a process termed “type II programmed cell death”^14^. Recent studies demonstrated that autophagy is closely associated with the development of many chronic pathological conditions such as cancer, inflammation, neurodegenerative diseases, and metabolic disorders^15^,^16^. This suggests that autophagy plays a significant role in regulating many long-term physiological processes involved in cell fate determination.

We previously hypothesized that autophagic cell death plays a key role in aminoglycoside-induced delayed ototoxicity. Consistent with this, GM treatment induced autophagosome formation in a time-dependent manner that was accompanied by cell death. Therefore, we hypothesized that autophagy might be an important regulator of aminoglycoside drug-induced ototoxicity. The purpose of this study was to identify the mechanisms of GM-induced delayed ototoxicity by exploring the role of autophagy using *in vitro* and *in vivo* studies, and also develop appropriate materials that could protect against GM-induced hearing loss.

## Results

### GM induces auditory cell death by stimulating autophagosome accumulation in a time-dependent manner

**Figure 1:**
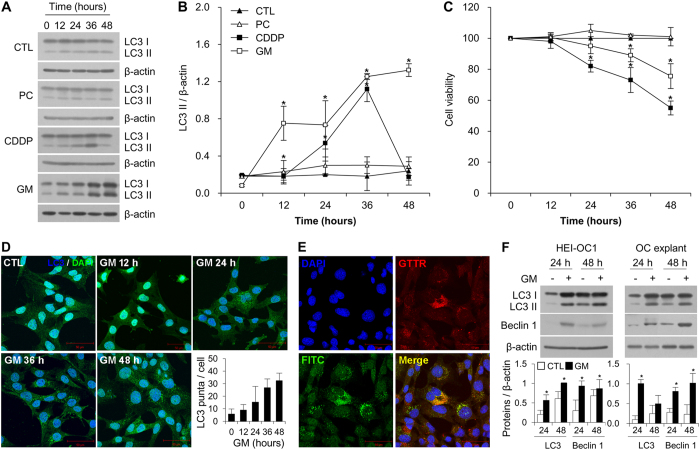
GM-induced autophagosome accumulation in auditory cells. (**A**) Western blot analysis of LC3 in HEI-OC1 cells treated with penicillin (PC), cisplatin (CDDP) and gentamicin (GM) for the indicated time periods. Equal protein loading was verified using β-actin expression. (**B**) Densitometric analysis of the blots showing the ratios of LC3 to β-actin (*n* = 3). (**C**) Cell viability was quantified using MTS assays. Viability of control cells was set to 100%, and viability relative to the control is shown. Data are presented as mean ± S.D (*n* = 3). (**D**) Representative fluorescence images of LC3 (green) and DAPI (blue) staining in HEI-OC1 cells after treatment with GM (5 mM) for various times. The white square in the upper image indicates the area magnified in the lower panel. Bar graph showing the mean number of LC3 puncta per cell ± S.D. (**E**) Representative fluorescence images of the colocalization of LC3 (green) with GTTR (red) in HEI-OC1 cells after treatment with GTTR. Fluorescence microscopy analyses were carried out in triplicate for each sample. (**F**) Western blotting analysis of LC3 and Beclin 1 expression in GM-treated and untreated HEI-OC1 cells and OC explants after 24 and 48 h. Western blot data from three independent experiments were quantified and normalized to β-actin levels. Statistical analyses were performed using Mann Whitney U tests (*P < 0.05).

We investigated the autophagic changes induced in House Ear Institute-Organ of Corti 1 (HEI-OC1) auditory cells by the ototoxic drugs GM and cisplatin, as well as the non-ototoxic drug penicillin, using western blotting and cell viability assays. At the initiation of autophagy the cytosolic form of microtubule-associated protein 1 light chain 3 (LC3-I, 18 kDa) is conjugated to phosphatidylethanolamine to form LC3-phosphatidylethanolamine conjugate (LC3-II, 16 kDa), which is then incorporated into autophagic membranes. Therefore, LC3-II is used as a marker of autophagosome formation^17^. As expected, penicillin did not affect LC3-II expression and the survival of HEI-OCI cells. However, culturing auditory cells with 7 μM cisplatin for 48 h increased the levels of LC3-II abruptly between 12 and 36 h; levels then decreased significantly at 48 h concomitant with cell death. In contrast, treatment with 5 mM GM induced a continuous increase in LC3-II expression until 48 h, and also induced cell death (Fig. 1A–C). Immunocytochemistry revealed a time-dependent increase in punctuated expressed LC3-II in cells treated with GM (Fig. 1D). The level of autophagosome formation was higher in gentamicin-conjugated Texas Red (GTTR)-expressing cells compared with non-expressing cells, and LC3-II co-localized with GTTR (Fig. 1E).

We used an *ex vivo* organotypic system to confirm these observations since it provides a better model for GM ototoxicity than the *in vitro* system, which requires a relatively high dose of GM to induce hair cell death. Coiled-coil, myosin-like Bcl-2 interacting protein (Beclin 1) and its binding protein class III phosphoinositide 3-kinase (PI3K) are essential for the initiation of autophagosome formation. The upregulation of LC3-II and Beclin 1 was observed 24 and 48 h after GM treatment in both HEI-OC1 cells and organ of Corti (OC) explants, which was accompanied by decreased cell viability (Fig. 1F). These results suggest that the accumulation of autophagosomes in auditory cells is closely related to GM-induced cell death.

### Autophagy modulation by pharmacological agents affects GM-induced ototoxicity

**Figure 2:**
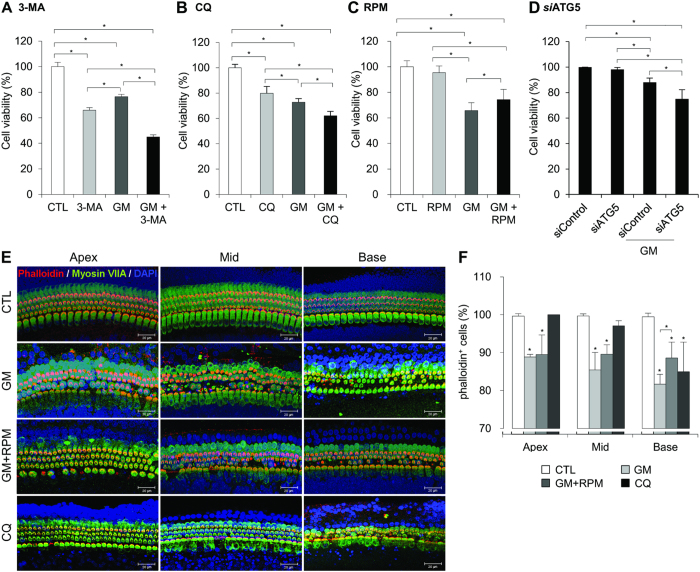
Modulating autophagy using pharmacological agents affects GM-induced ototoxicity. Cells were either treated with the autophagy inhibitor 3-MA (5 mM) (**A**), autophagy inhibitor CQ (25 μM) (**B**), or autophagy inducer RPM (0.5 nM) (**C**) during exposure to 5 mM GM for 48 h. (**D**) Cells were transfected with *ATG5* siRNA or control siRNA for 24 h and were subsequently treated with or without 5 mM GM for 48 h. Cell viability was quantified using MTS assays. The relative percent cell viability was calculated relative to the viability of control HEI-OC1 cells. The bar graph presented as mean ± S.D (*n* = 3). Statistical analyses were performed using Mann Whitney U tests (*P < 0.05). (**E**) Confocal images of phalloidin (red), myosin VIIa (green), and DAPI (blue) triple-labeled cells from the apex to the base in OC explants treated with dH_2_O (first column), 50 μM GM (second column), 50 μM GM + 0.5 nM RPM (third column), and 25 μM CQ (fourth column). (**F**) Quantification of the hair cell density in the apex, mid and base of OC explants. Data are presented as mean ± S.D (*n* = 3), and statistically significant differences were identified using Mann Whitney U tests (*P < 0.05).

The class III PI3K inhibitor 3-methyladenine (3-MA) is a potent autophagy inhibitor that blocks autophagosome formation. Unlike 3-MA, the autophagy inhibitor chloroquine (CQ) prevents the fusion of autophagosomes with lysosomes and inhibits the lysosomal degradation of proteins by neutralizing vacuolar pH at the late stage of autophagy^18^. When HEI-OC1 cells were cultured with GM and 3-MA or CQ for 48 h, GM-induced cell death was enhanced significantly by 3-MA or CQ (Fig. 2A,B). However, toxic effects of 3-MA and CQ on HEI-OC1 cells were also observed in the absence of GM. Although cell death was detected even when cells were treated with the minimum inhibitory concentration of 3-MA and CQ for 24 h, 3-MA and CQ also enhanced GM-induced cell death. These results suggest that autophagic flux is required for cell maintenance under both normal and pathological conditions. To investigate the effects of autophagic flux on the viability of auditory cells, we used an inhibitor of mammalian target of rapamycin (mTOR), rapamycin (RPM) to induce autophagy. As shown in Fig. 2C, RPM significantly prevented GM-induced cell death. Furthermore, siRNA-mediated knockdown of *ATG5* gene, which is required for autophagosome formation^19^, also decreased cell viability compared to the nonspecific scrambled siRNA (*si*Control) in GM-treated cells (Fig. 2D).

To confirm the effects of RPM on hair cell viability, we cultured *ex vivo* OC explants with 50 μM GM and 100 pM RPM for 48 h. Generally, the first row of outer hair cells (OHCs) was damaged first by GM, followed by the second and third row OHCs^20^ along a base-to-apex gradient. Next, phalloidin-myosin VIIa staining was used to count hair cells at the base, mid, and apex. A normal pattern of three rows of OHCs and a single row of inner hair cells (IHC) was observed in the control explants. However, treatment with 50 μM GM caused derangement of the stereocilia and loss of hair cells (11.2 ± 0.7, 14.6 ± 4.6, and 18.3 ± 2.6% for the apex, mid, and base turn, respectively). These effects of GM were attenuated by RPM (10.5 ± 5.2, 10.4 ± 2.5, and 12.7 ± 4.2% for the apex, mid, and base turn, respectively; Fig. 2E,F). Consistent with the *in vitro* data, CQ had cytotoxic effects and caused the loss of stereocilia on basal turns. Taken together, these data suggest that enhanced autophagic flux could prevent GM-induced auditory cell death.

### GM suppresses autophagic flux by decreasing the fusion of autophagosomes with lysosomes

**Figure 3:**
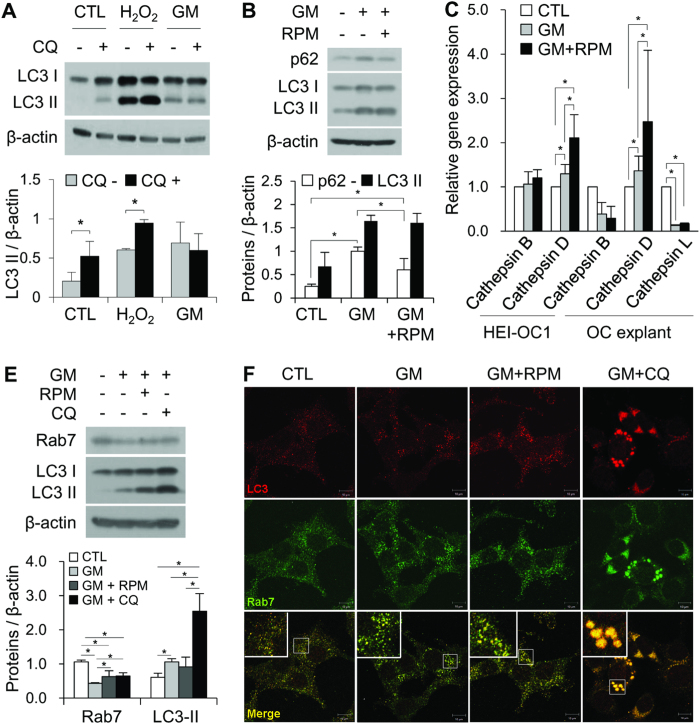
The autophagy inducer RPM enhances autophagic flux in auditory cells. (**A**) Western blotting analysis of LC3 expression in HEI-OC1 cells incubated in normal medium or medium containing GM (5 mM, 24 h) or H_2_O_2_ (100 μM, 2 h) in the absence or presence of 50 μM CQ for 4 h. (**B**) Western blotting analysis of p62 and LC3 in untreated HEI-OC1 cells and cells treated with 5 mM GM alone or 5 mM GM with 0.5 nM RPM. Data from three independent experiments were quantified and normalized to β-actin. Statistical analyses were performed using Mann Whitney U tests (*P < 0.05). (**C**) The levels of *cathepsin B* and *cathepsin D* mRNA were measured in HEI-OC1 cells and OC explants in the control, 5 mM GM, and 5 mM GM with 0.5 nM RPM groups. Expression was normalized to *GAPDH* levels, and the results are expressed as fold-change compared with control. The bar graph presented as mean ± S.D (*n* = 3). (**D**) Western blotting and (**E**) fluorescence microscopy analyses of Rab7 and LC3 in untreated HEI-OC1 cells and cells treated with 5 mM GM alone, 5 mM GM with 0.5 nM RPM or 5 mM GM with 25 μM CQ. Densitometric analysis of the corresponding blot is shown below the western blot. Statistical analyses were performed using Mann Whitney U tests (*P < 0.05, *n* = 3).

There are two possible mechanisms to explain the GM-induced autophagosome accumulation: the increased activation of autophagic flux and the inhibition of autophagosome degradation. To differentiate these two mechanisms, we assessed the changes in LC3-II accumulation in untreated cells and cells treated with hydrogen peroxide (H_2_O_2_) or GM in the presence or absence of CQ. Many studies have shown that autophagic flux is upregulated in response to oxidative stress^21^. As shown in Fig. 3A, LC3-II accumulation was higher in CQ-treated cells compared with untreated cells, even in the presence of H_2_O_2_. Similar to CQ, treatment with GM alone led to the upregulation of LC3-II; however, the concomitant use of GM and CQ did not induce a further increase in LC3-II levels (Fig. 3A). Therefore, we predicted that GM, like CQ, could suppress autophagic degradation activity in lysosomes. Data revealed increased levels of p62/SQSTM1, a specific substrate that binds to LC3 to facilitate the degradation of ubiquitinated proteins via the autophagy-lysosomal pathway. The levels of p62/SQSTM1 were significantly lower in cells co-treated with GM and RPM compared with those treated with GM alone (Fig. 3B). Next, we analyzed the expression of the major intracellular lysosomal cysteine proteases cathepsin B, D, and L using reverse-transcriptase polymerase chain reaction (qRT-PCR). As shown in Fig. 3C, the levels of *cathepsin D* mRNA were higher in HEI-OC1 cells and organ of Corti explants treated with both RPM and GM compared with cells and explants treated with GM alone (2.11 ± 0.52 vs. 1.30 ± 0.21, and 2.47 ± 1.61 vs. 1.37 ± 0.33, respectively). In contrast, RPM did not significantly affect *cathepsin B* or *cathepsin L* expression (*P* > 0.05).

Rab is a member of the small GTP binding protein family that is involved in the late autophagic processes such as endosome trafficking and lysosome biogenesis. Rab7 levels were decreased in GM-treated HEI-OC1 cells compared with control cells (Fig. 3E). Previous studies demonstrated that Rab7 is required for the maturation of autophagic flux^22^. In addition, Gutierrez *et al*. reported that the overexpression of a Rab7 dominant negative mutant (Rab7T22N) led to the accumulation of large autophagosomal vacuoles, probably by disrupting normal progression of the autophagic pathway^23^. Consistent with these reports, this study revealed that there was a significant increase in the size of vesicles in cells treated with GM or CQ (Fig. 3F). These results suggest that autophagosome accumulation was caused by GM-induced defects in the late endosome-lysosome step of autophagy.

### Autophagic vacuole accumulation is increased in auditory hair cells from GM-treated animals

**Figure 4:**
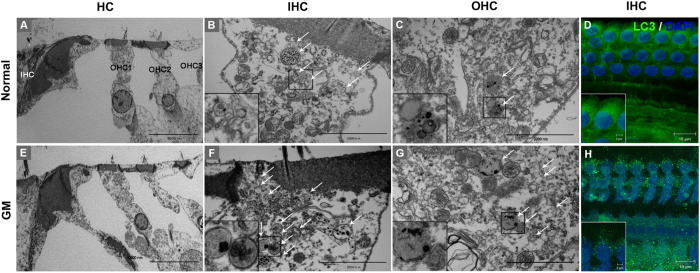
GM induces autophagosome accumulation in cochlear hair cells. TEM images of autophagosomes in GM-injected (**E**–**G**) and uninjected (**A**–**C**) SD rats. Low magnification TEM images show cross sections across the organ of Corti; OHC, outer hair cell; IHC, inner hair cell (**A**,**E**). Representative TEM micrographs of autophagic vacuoles in outer hair (**B**,**F**) and inner hair cells (**C**,**G**). White arrows indicate autophagic vacuole-like structures. Scale bar, 10 μm (**A**,**E**) and 2 μm (**B**,**C**,**F** and **G**). (**D**,**H**) Representative fluorescence images of LC3 (green) and DAPI (blue) staining in hair cells from GM-injected (lower panel) and uninjected (upper panel) SD rats. Scale bar, 10 μm.

Sprague Dawley (SD) rats were injected intraperitoneally with GM (220 mg/kg) once per day for 5 days to induced ototoxicity. The autophagic response in auditory hair cells within the organ of Corti was then analyzed using transmission electron microscopy (TEM) and immunohistochemistry. Autophagic vacuoles were observed in the inner and outer hair cells of normal rats (Fig. 4A–C). However, the number of autophagic vacuoles in the inner and outer hair cells increased 10 days after GM injection (Fig. 4E–G). Disorganized structures and the loss of cristae in mitochondria were observed in GM-injected animals as the number of autophagic vacuoles increased. In addition, immunohistochemical analysis of the organ of Cortis revealed that LC3 staining was more intense and condensed in GM-injected animals compared with control animals (Fig. 4D,H).

### Enhanced autophagic flux induces otoprotective effects against GM in rats

**Figure 5:**
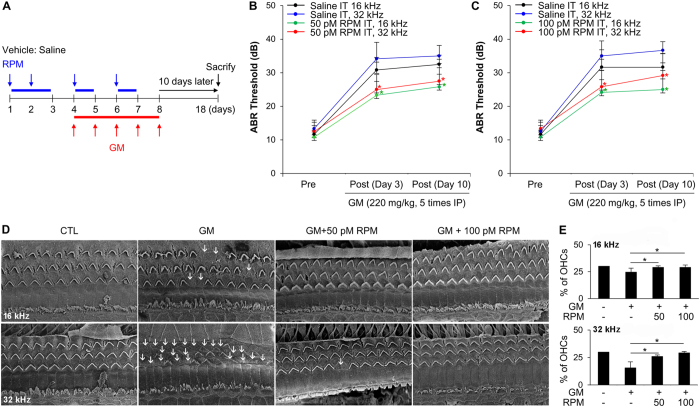
Activation of autophagic flux by RPM protects against GM-induced hearing loss. (**A**) Schematic diagram of the *in vivo* experimental procedures using SD rats. (**B**,**C**) ABR measurements at 16 and 32 kHz before (pre) and after (post-day 3 and post-day 10) I.T. injection with RPM in the left ear and normal saline in the right ear of rats injected with GM (220 mg/kg, intraperitoneally). The left side of the graph shows the results for 50 pM RPM (**B**), and the right side of the graph shows the results for 100 pM RPM (**C**). (**D**) SEM images of outer hair cell stereocilia bundles in the organ of Corti from 16 kHz (upper image) and 32 kHz region (lower image). White arrows indicate the loss of stereocilia. (**E**) Bar graphs showing the number of stereocilia in the middle turn and basal turn. Statistical analyses were performed using Mann Whitney U tests (*P < 0.05 vs. GM alone, *n* = 3).

SD rats were injected intraperitoneally with GM and then treated by intratympanic injection with RPM (50 and 100 pM) or CQ (6 and 20 μM) in the left ear four times per week to manipulate autophagy (Fig. 5A). The contralateral ear was injected with normal saline as a control. As shown in Supplementary Figure S1, LC3 accumulation was higher in RPM- or CQ-injected hair cells than in normal saline-injected hair cells in the organ of Corti. This suggests that inducing autophagy using RPM or inhibiting the autophagolysosomal pathway with CQ stimulates LC3 accumulation.

The hearing thresholds of auditory brainstem response (ABR) were determined at 16 and 32 kHz (mid-basal turn of the cochlea), because GM-induced hearing loss caused by hair cell damage or death, particularly at the mid-basal turn. The mean hearing thresholds at 16 and 32 kHz before GM treatment was 11.7 ± 3.0 and 13.3 ± 2.2 dB, respectively; there was no significant difference among groups (Fig. 5B,C). On the third day after GM treatment, marked hearing loss was detected in rats injected with GM-only, with thresholds increasing to 31.2 ± 4.5 dB at 16 kHz and 34.6 ± 4.7 dB at 32 kHz. However, the hearing thresholds were significantly better in the contralateral ears of rats in the RPM group (24.2 ± 3.8 dB at 16 kHz and 25.0 ± 4.5 dB at 32 kHz in 50 pM RPM-injected ears; 23.3 ± 4.1 dB at 16 kHz and 25.8 ± 4.9 dB at 32 kHz in 100 pM RPM-injected ears; *P* < 0.05) compared with the GM-only injected ears (Fig. 5B,C). These statistically significant differences remained after 10 days of GM treatment.

Scanning electron microscopy (SEM) analysis of representative segments of the mid-basal turn showed that the number of hair cells with normal stereocilia was unaffected in the control group and reduced in GM alone-injected ears. However, only a small number of hair cells were damaged in ears co-injected with RPM (50 or 100 pM) and GM (Fig. 5D,E). The difference in the number of intact stereocilia between these two groups was statistically significant at both frequencies (*P* < 0.05; Fig. 5D,E).

## Discussion

Apoptosis has been the main focus of studies into aminoglycoside antibiotic-induced ototoxicity to date, including JNK activation, caspase 9 and caspase 3 cleavage, and mitochondrial cytochrome C release. However, apoptosis is thought to be the final step in aminoglycoside-induced toxicity. Recent studies showed that GM-induced hair cell damage can occur through a caspase-independent pathway and that inhibiting caspases did not completely prevent hair cell death^24^. In addition, aminoglycoside antibiotics induce delayed toxic effects compared with other ototoxic drugs. Therefore, we hypothesized that GM-induced ototoxicity might be differentially regulated in hair cells and that some specific mechanisms may be related to GM-induced cell death.

Taylor *et al*.^25^ reported diverse mechanisms of cell death in auditory hair cells^25^. Aminoglycoside antibiotics induced autophagy-like features, including cup-shaped double membrane structures called autophagosomes, as well as apoptotic features such as nuclear fragmentation and chromatin condensation^25^. In this regard, there have been reports on the autophagic responses of hair cells to environmental insults and aging^26^. Fang *et al*. found that RPM protects against the otoprotective effects of cisplatin, probably by inducing autophagy^27^. Yuan *et al*. also suggested that autophagy plays a protective role against noise-induced hearing loss by attenuating oxidative stress, as indicated by products of lipid oxidation and protein nitration levels^28^. Considering that autophagy is a slower process than apoptosis^29^, it is possible that autophagy may play a more significant role in aminoglycoside-induced cell death than cisplatin or noise. However, the mechanism by which autophagy affects auditory cell death during ototoxic damage is unclear.

Although autophagy is considered a cell survival mechanism against cellular stress, increasing evidence has suggested potential links between autophagy and several pathophysiology states including cancer, neurological disorders, aging, and infection. Most studies have demonstrated that autophagy is harmful in these states, mainly via defective or excessive autophagy. The data in this study also showed that GM could attenuate autophagy flux by suppressing late stage autophagosome degradation and thereby play a role in causing hearing loss.

Several lines of evidence suggest that autophagic flux plays an important role in pathological processes. Autophagic flux is determined by the equilibrium between autophagosome formation and autophagosome clearance by lysosomes. Thus, defects in the early or late steps of autophagy cause autophagic dysfunction, which subsequently induces autophagic cell death^30^. In neuronal cells, glucose reperfusion after glucose deprivation impaired autophagic flux: p62/SQSTM1 levels increased, indicating suppressed autophagy, and LC3-II levels and cell death were augmented^30^. In addition, Gonzalez-Rodriguez *et al*. recently reported that autophagic flux is impaired in the livers of patients with hepatic and non-alcoholic fatty liver disease (NAFLD)^31^. Increased mTOR and S6K1 phosphorylation were observed in Huh7 human hepatic cells treated with palmitic acid, which was accompanied by the accumulation of p62, an increased LC3-II/LC3-I ratio, and cell death. Furthermore, activating autophagy using RPM attenuated the progression of NAFLD.

In this study, GM stimulated autophagosome formation as well as the upregulation of LC3-II and Beclin 1 in HEI-OC1 cells. The increased LC3-II levels could reflect either increased autophagosome production at the early stage of autophagy or inhibited autophagosome clearance during late-stage autophagy^32^. To differentiate between these possibilities, we examined the change in autophagic flux using the autophagy inhibitor CQ, which inhibits lysosome-mediated autophagosome degradation. Interestingly, no further increase in LC3-II levels was observed when CQ was added to GM-treated HEI-OC1 cells. This suggests that the increase in LC3-II levels in GM-treated cells was not caused by increased autophagic flux, but rather by inhibited lysosome-mediated autophagosome degradation. If GM induced autophagic flux, the LC3-II levels would have increased further when autophagosomal degradation was inhibited using CQ.

The altered levels of Rab7 and cathepsin B and D observed in HEI-OC1 cells in this study support the hypothesis that autophagic flux was arrested at a late stage of autophagy following GM treatment. Rab7 is a member of the Rab family of Ras-related GTP-binding proteins and is required for transport from early to late endosomes/lysosomes and for lysosome biogenesis^33^. Recent studies demonstrated that Rab7 plays a key role in the maturation of late autophagic vesicles and fusion with lysosomes^23^,^33^. The overexpression of a Rab7 dominant-negative mutant induced autophagosome accumulation, increased the size of monodansylcadaverine-labeled autophagic vacuoles, and impaired the autophagic degradation of long-lived proteins^33^. Our data demonstrated that Rab7 was also downregulated by GM exposure in HEI-OC1 cells.

Pharmacologic or genetic manipulations have been used to understand the role of autophagy. In particular, the application of pharmacological autophagy inducers (RPM, resveratrol, metformin, lithium, trehalose, and spermidine) may be useful therapeutic strategies in conditions associated with insufficient autophagy. RPM, a specific inhibitor of mTOR, is used most commonly to induce autophagy and its beneficial effects are exerted through various pathways. Yin *et al*.^34^ reported that RPM attenuated palmitate-induced adipocyte dysfunction by stimulating autophagy, which in turn reduced endoplasmic reticulum stress by altering eIF2α phosphorylation, ATF4, CHOP, and JNK phosphorylation^34^. Nacarelli *et al*. also reported that RPM contributed to reduced mitochondrial and cellular ROS production and increased cell viability. Consistent with this, our previous study showed that activating autophagic flux using RPM decreased cell death in GM-treated cell cultures and animal models^35^.

In this study, adding RPM to GM-treated cells led to the upregulation of Rab7 protein and increased the subcellular colocalization of Rab7 and GTTR. In addition, RPM activated the lysosomal cysteine proteases cathepsin B and D, which are important for autophagic degradation^36^. Therefore, RPM-induced autophagosome/lysosome-mediated protein degradation appears to have protective effects against GM-induced ototoxicity. However, the exact molecular mechanism by which RPM induces cell survival at the late stage of autophagosome degradation remains unclear.

Ototoxic animal models have been used to study hearing loss and for drug screening. Aminoglycoside-induced ototoxic animal models are usually evaluated several days (at least 1 week) post-injection^37^ because aminoglycoside antibiotics induce delayed ototoxicity in humans^4^. This study verified that the intraperitoneal injection of SD rats with 220 mg/kg GM increased the ABR threshold and promoted the loss of stereocilia after 10 days. In addition, consistent with our *in vitro* results, co-treatment with RPM (50 or 100 pM) attenuated the GM alone-induced increase in ABR threshold and the loss of stereocilia. Therefore, we assessed whether the effects of the autophagy inhibitor CQ were opposite those of RPM. Drug toxicity-induced hearing loss was observed after treatment with CQ alone (Fig. S2), which is consistent with both the *in vitro* and *ex vivo* data (Fig. 2). Previous studies described sensorineural hearing loss, tinnitus, and a sense of imbalance after CQ treatment^38^, suggesting that autophagy plays an essential role in maintaining normal auditory cellular functions.

The exact mechanism of GM-induced delayed ototoxicity has not been fully elucidated. The delayed toxicity may occur after the accumulation of toxic chemicals in cells in response to GM. It is also possible that the delayed ototoxicity may be dependent on pathways different from those activated by other ototoxic drugs such as cisplatin. This study suggests that autophagy may play dual roles in cell survival at the early stage and cell death in the late stage of autophagy after exposure to GM. Thorburn *et al*. reported that trehalose, a novel autophagy enhancer, “slowed” death after tumor necrosis factor-related apoptosis inducing ligand (TRAIL) treatment by promoting inefficient mitochondrial outer membrane permeabilization (MOMP). This in turn activated the mitochondrial apoptotic pathway. The authors suggested that autophagy could regulate the timing of MOMP in apoptosis^29^. Therefore, autophagy may be a key pathway in GM-induced delayed toxicity.

**Figure 6:**
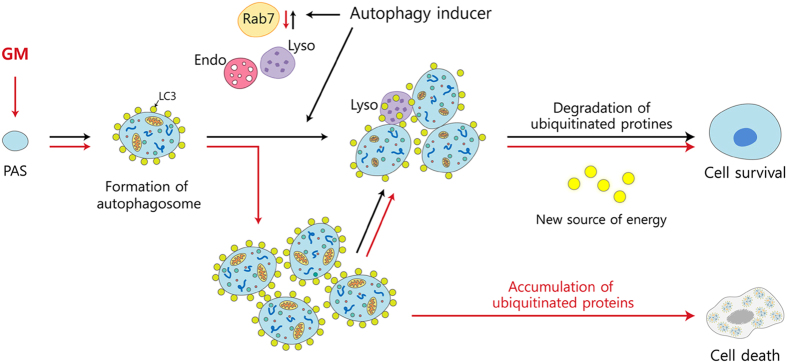
Schema of the proposed process underlying the regulatory role of autophagy in gentamicin-induced hair cell death.

In summary, we demonstrated that GM contributes to autophagic cell death by disrupting autophagic flux in auditory cells. In addition, the pharmacological enhancement of autophagic flux improves auditory cell survival in response to GM toxicity both *in vitro* and *in vivo* (Fig. 6). This deterioration of autophagy may be closely related to GM-induced delayed ototoxicity.

## Materials and Methods

### Experimental design of HEI-OC1 cells and OC explants

HEI-OC1 cells, conditionally immortalized cochlear epithelial cells, were maintained in Dulbecco’s modified Eagle medium (DMEM; Gibco-BRL) supplemented with 10% fetal bovine serum (FBS; Gibco-BRL, 16000-044) at 33 °C with 10% CO_2_. OC explants from Sprague-Dawley rats at postnatal day 7 were isolated as previously described^39^. Explants were maintained in DMEM including 10% FBS and 0.06 mg/ml penicillin at 37 °C with 5% CO_2_. GM (Sigma-Aldrich, G1264) was prepared in sterile dH_2_O. RPM (LC Laboratories, R-5000) was prepared in dimethyl sulfoxide (DMSO). 3-MA (Sigma-Aldrich, M9281) and CQ (Sigma-Aldrich, C6628) were prepared in sterile dH_2_O. Controls for each test compound used the respective solvent. Hydrogen peroxide (H_2_O_2_; Sigma-Aldrich, 516813) was used at 1 mM as a positive control for autophagy.

### Conjugation and purification of GTTR

GM sulfate (50 mg/ml in 100 mM K_2_CO_3_, pH 9; Sigma-Aldrich, G1264) and Texas Red succinimidyl ester (2 mg/ml in N,N-dimethyl formamide; Molecular Probes, T-353) were mixed at a ratio of 300:1 for 3 days. The reaction mixture was diluted in 5% glacial acetic acid (GAA). Unconjugated GM and Texas red were eluted with 5% GAA and methanol, respectively. After purification through a C-18 column, the probes were stored in the dark at −20 °C.

### Preparation for animal studies

The Ajou University School of Medicine Institutional Animal Care and Use Committee approved the surgical procedures, which were performed in accordance with guidelines on the care and use of animals for experimental procedures. All efforts were made to minimize the number of animals used and their suffering. Sprague-Dawley rats (SD rats; male, 7 weeks old, 200–250 g) were purchased from Orient Bio Inc. Twelve rats were divided into group 1 (n = 6) and group 2 (n = 6). GM at a dose of 220 mg/kg was administered intraperitoneally for five consecutive days. Rats were intraperitoneally anesthetized with Zoletil 50 (0.1 cc/100 g; Virbac Laboratoire) and Rompun 2% (0.02 cc/100 g; Bayer Korea). They then received intratympanic (I.T.) injection of RPM (50 pmol or 100 pmol in 20 μl for group 1 and group 2, respectively) in the left ear every other day (two times as pretreatment, three times as co-treatment with GM, and two times as post-treatment). The contralateral ears were injected with normal saline. Hearing function was evaluated before the first RPM administration and on days 3 and 10 after the last GM administration. Cochleae were harvested for morphological evaluation (Fig. 5A).

### Auditory brainstem response (ABR) test

The auditory brainstem response (ABR) was tested with the BioSig 32 system (Tucker-Davis Technologies, Gainesville, FL, USA) as previously described^40^. The threshold was defined as the lowest stimulus level at which a clear waveform was visible in the evoked trace.

### Scanning electron microscopy (SEM)

Rats were decapitated quickly after anesthetization, and the temporal bones were harvested. The perilymphatic space was perfused with 4% glutaraldehyde. Each specimen was then placed in the same glutaraldehyde solution overnight at 4 °C. After 3 rinses with phosphate-buffered saline (PBS), the end-organ surfaces were prepared for SEM. Samples were serially dehydrated in 50%, 70%, 90%, 95%, and 100% acetone. Each specimen was exposed to hexamethyldisilazane, air dried, and then placed on a stub for gold-sputter coating. Photographs were taken with a JSM-5410 LV SEM camera (Jeol). The number of hair cells in the organ of Corti was analyzed as previously described^40^.

### Small interfering RNA (siRNA)-induced gene silencing

The siRNA were synthesized by Genolution Parmaceuticals, Inc. (Seoul, South Korea). The target sequences for siRNAs were as follows: 5′-CUGUCUUUGCUGUUACGUU-3′ and 5′ AACGUAACAGCAAAGACAG-3′ for *ATG5*, the nonspecific negative control (*si*Control). The siRNA were transfected into HEI-OC1 cells using the lipofectamine RNAi Max (Invitrogen, Carlsbad, CA, USA).

### Transmission electron microscopy (TEM)

Tissues were fixed in cold 2.5% glutaraldehyde in 0.1 M PBS, pH 7.4. After an overnight incubation, specimens were post-fixed in 1% osmium tetroxide, dehydrated in graded alcohols, and embedded in Spurr’s epoxy resin. After uranyl acetate and lead citrate staining, specimens were viewed with a transmission electron microscope (EM 902 A; Carl Zeiss MicroImaging GmbH).

### Western blot

Cells were lysed in RIPA buffer (25 mM Tris·HCl pH 8, 150 mM NaCl, 1% NP-40, 0.5% sodium deoxycholate, 0.1% sodium dodecyl sulfate (SDS)) with a protease inhibitor cocktail (GenDEPOT, P3100). After extracting the proteins by centrifugation (13,000 rpm, 30 min), equal amounts of protein were loaded onto each lane of a gel, separated by SDS-polyacrylamide gel electrophoresis, and then transferred onto a polyvinylidene difluoride (PVDF; Millipore, IPVH00010) membrane using an electroblotting apparatus. Membranes were blocked for 1 h with 5% skim milk in PBS + 0.05% Tween 20. Subsequently, the membranes were probed with a primary antibody overnight at 4 °C. The primary antibody was detected using horseradish peroxidase-labeled secondary antibodies and a WEST-ZOL Plus western blotting detection system (iNtRON Biotechnology, 16024). Proteins levels were quantified with densitometry analysis using ImageJ. The following primary antibodies were used: anti-LC3 (Sigma-Aldrich, L7543), anti-Beclin 1 (Santa Cruz, sc-11427), anti-phospho SAPK/JNK (Santa Cruz, sc-6254), anti-α-Tubulin (Santa Cruz, sc-32293), anti-Rab7 (Cell Signaling, #9367), anti-PARP (Cell Signaling, #9542), anti-caspase 3 (Cell Signaling, #9662), anti-total SAPK/JNK (Cell Signaling, #9252), and anti-β-actin (Cell Signaling, #4970).

### Quantitative reverse-transcriptase polymerase chain reaction (qRT-PCR)

Total RNA was isolated from the cells using an Easy-BLUE RNA extraction kit (iNtRON Biotech, 17061). Standard RT was performed using an amfiRivert cDNA Synthesis kit (GenDEPOT, R5101) according to the manufacturer’s instructions. qRT-PCR measurements were performed using the ABI Prism 7000 Sequence Detection System (Applied Biosystems) and a SYBR Green I qPCR kit (Takara, RR420) according to the manufacturers’ instructions. The cDNA was amplified with the following primers: 5′-GAAGAAGCTGTGTGGCACTG-3′ and 5′-GTTCGGTCAGAAATGGCTTC-3′ for mouse cathepsin B, 5′-AGGTGAAGGAGCTGCAGAAG-3′ and 5′-ATTCCCATGAAGCCACTCAG-3′ for mouse cathepsin D, and 5′-AACGGGAAGCCCATCACC-3′ and 5′-CAGCCTTGGCAGCACCAG-3′ for mouse GAPDH and 5′-GATCGCACAGGTCTTTAAGAA -3′ and 5′-CATCGACGTTGGGTAGAC AC-3′ for rat cathepsin B, 5′-ACACTGTGTCGGTTCCATGT -3′ and 5′-TGCGATGAATACGACTCCAG -3′ for rat cathepsin D, and 5′-CAA AGACCGGAACAACCACT -3′ and 5′-CCTTCGGATGTAGTGTCCGT-3′ for rat cathepsin L and 5′-GTTACCAGGGCTGCCTTCTC -3′ and 5′-GGGTTTCCCGTTGATGACC-3′ for rat GAPDH. With normalization to GAPDH and GUSB, the relative gene expression was analyzed using the comparative threshold cycle (CT) method (Applied Biosystems). The expression of the genes of interest was expressed as fold change over control.

### Immunocytochemistry

For immunocytochemistry, cells were grown on round glass coverslips (Marienfeld). Cells were fixed for 20 min at room temperature with 4% paraformaldehyde, permeabilized in 0.2% Triton-X 100/PBS for 10 min, and blocked with 1% bovine serum albumin (BSA; GenDEPOT, A0100-100) in PBS. Primary antibodies were applied at 4 °C overnight. The cells were washed and labeled with fluorescein isothiocyanate (Jackson ImmunoResearch Laboratories, 111-095-003) or cyanine 3 (Jackson ImmunoResearch Laboratories, 115-165-003) for 1 h. Nuclei were stained with 1 μg/ml 4′,6′-diamidino-2-phenylindole (DAPI; Invitrogen, D1306) in PBS for 1 min at room temperature. The cells were visualized with a Zeiss LSM 700 confocal microscope (Carl Zeiss Microscopy) or an AxioVision LE 4.5 microscope (Carl Zeiss MicroImaging Inc.).

### Immunohistochemistry

After isolation, cochlea was fixed with 4% paraformaldehyde and kept overnight at 4 °C. After decalcification with Calci-Clear Rapid solution (National Diagnostics, HS-105) for 5 days, the organ of Corti was removed. Tissue were mounted on gelatin-coated slides and blocked with 1% BSA in PBS containing 0.2% Triton X-100 (PBST) for 1 h. After washes with PBST, tissues were incubated with primary antibody for 24 h at 4 °C, washed in PBST, and incubated with the corresponding secondary antibody for 1 h. The nuclei were counterstained with DAPI (1:10,000). The immunostained cells were observed using a Zeiss LSM 700 confocal microscope (Carl Zeiss Microscopy).

### 3-(4,5-Dimethylthiazol-2-yl)-5-(3-carboxymethoxyphenyl)-2-(4-sulfophenyl)-2H-tetrazolium (MTS) assay

Cell viability was determined using the MTS assay (Promega, G3580). Auditory cells were seeded into 96-well plates at a density of 3 × 10^3^ cells/well. At the end of the incubation, MTS reagent was added to each well, and the cells were incubated for 4 h. Absorbance was measured using a microplate reader (Model 680; Bio-Rad) at 450 nm. All assays were performed at least three times in triplicate, and viability was normalized to that of the control.

### Statistical analysis

Data were expressed as mean ± s.d. Significant differences between two independent groups were analyzed by Mann-Whitney U test, and comparison of three or more groups were evaluated by Kruskal-Wallis tested using SPSS software (version 12.0, SPSS Inc.). P-values ≤ 0.05 were considered significant.

## Additional Information

**How to cite this article**: Kim, Y. J. *et al*. Autophagic flux, a possible mechanism for delayed gentamicin-induced ototoxicity. *Sci. Rep.* **7**, 41356; doi: 10.1038/srep41356 (2017).

**Publisher's note:** Springer Nature remains neutral with regard to jurisdictional claims in published maps and institutional affiliations.

## Supplementary Material

Supplementary Information
