# Supplementary material for: Autophagic flux, a possible mechanism for delayed gentamicin-induced ototoxicity
Source: Sci Rep. 2017 Feb 1;7:41356. doi: 10.1038/srep41356 (PMC5286410; doi:10.1038/srep41356)

Supplementary Information

Autophagic flux, a possible mechanism for delayed gentamicin-induced ototoxicity

Yeon Ju Kim[1], Chunjie Tian[2], Jangho Kim[3], Beomyong Shin[1,4], Oak-Sung Choo[1,5], You-Sun Kim[4] & Yun-Hoon Choung[1,5]

[1]Department of Otolaryngology, Ajou University School of Medicine, San 5 Woncheon-dong, Yeongtong-gu, Suwon 443-721, Republic of Korea

[2]Department of Otolaryngology, Dali Bai Autonomous Prefecture People's Hospital, Renminnan road 35, Dali, Yunnan 671000, China

[3]Department of Rural and Biosystems Engineering, Chonnam National University, Gwangju 500-757, Republic of Korea

[4]Department of Biomedical Sciences, BK21 Plus Research Center for Biomedical Sciences, Ajou University Graduate School of Medicine, San 5 Woncheon-dong, Yeongtong-gu, Suwon 443-721, Republic of Korea

[5]Department of Medical Sciences, Ajou University Graduate School of Medicine, San 5 Woncheon-dong, Yeongtong-gu, Suwon 443-721, Republic of Korea

[*]Author for correspondence: Yun-Hoon Choung, MD, DDS, PhD
Department of Otolaryngology, Ajou University School of Medicine, San 5, Wonchon-dong, Yeongtong-gu, Suwon 443-721, Republic of Korea.
Tel: +82-31-219-5263; Fax: +82-31-219-5264; E-mail address: yhc@ajou.ac.kr

Supplementary Figures

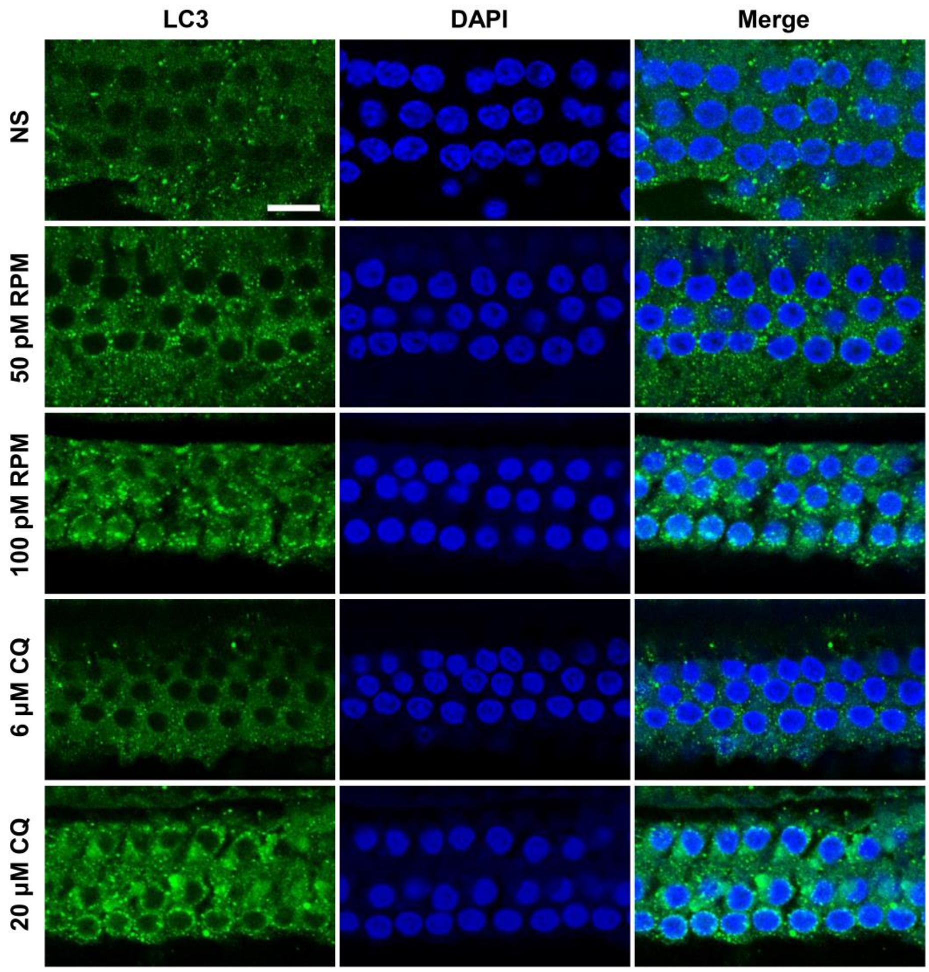

Supplementary Figure S1. Change in LC3 expression in the hair cells of the organ of Corti. Representative fluorescence images of LC3 (green, first column), DAPI (blue, second column), and their overlay (third column) from animals injected with normal saline (first row), 50 pM RPM (second row), 100 pM RPM (third row), 6 µM CQ (fourth row), or 20 µM CQ (fifth row) once every two days for a week. Scale bars: 10 µm.

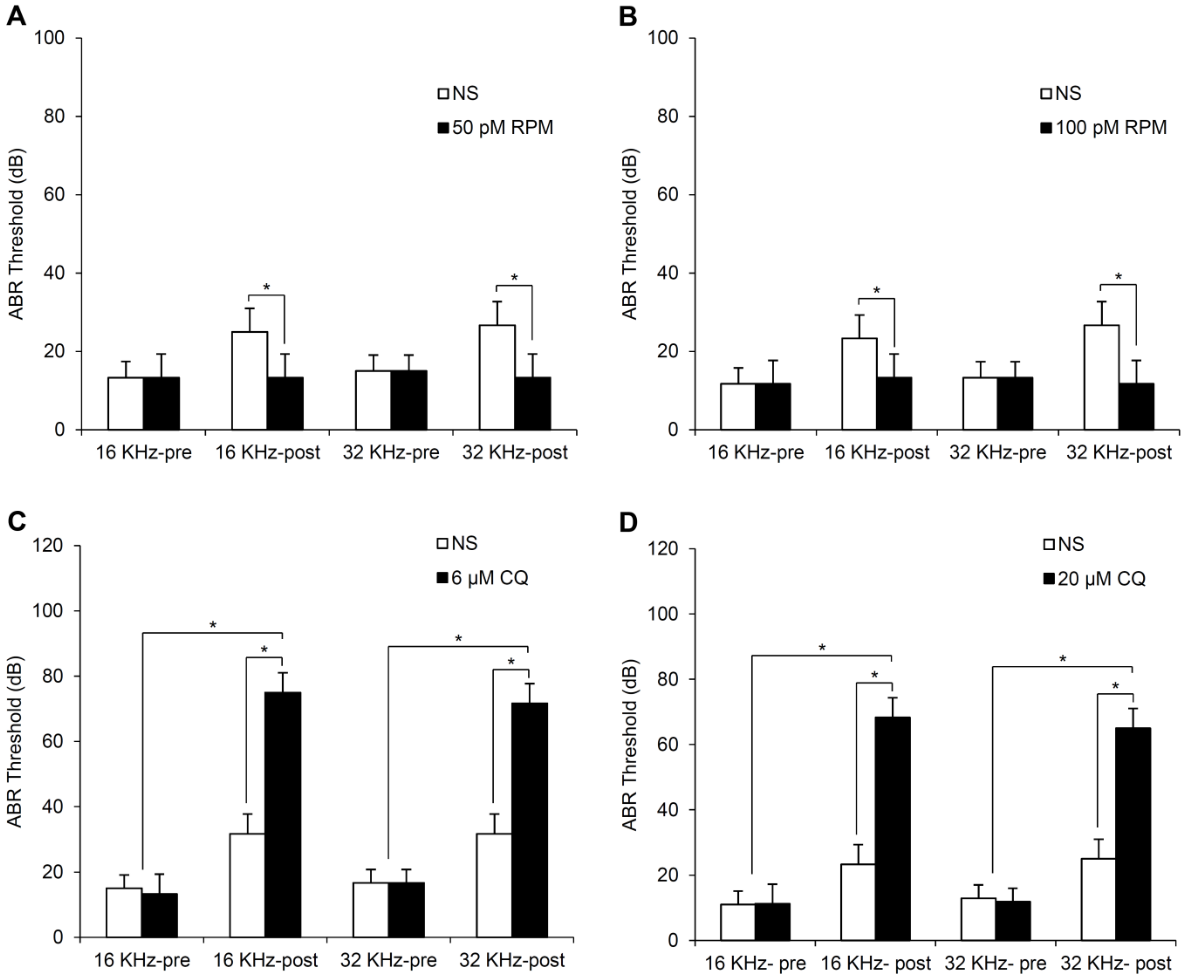

Supplementary Figure S2. ABR measurements after RPM and CQ treatment. The ABR threshold shift at 16 kHz and 32 kHz was measured before (white bars) and 3 days after (black bars) injection of 50 pM RPM **(A)**, 100 pM RPM **(B)**, 6 μM CQ **(C)**,or 20 μM CQ **(D)** into the left ear. As a control, the contralateral ear was injected with same volume of normal saline. Data were expressed as the mean ± SEM, with statistical analysis performed by Mann Whitney U test (*$P < 0.05$).

Western blot data

- To detect HRP (Horseradish peroxidase)-tagged antibodies in western blot, we used the manual film-development protocol using X-ray film, developer and fixer, not LAS digital imaging system. And western blot films were usually cropped after the confirm of the molecular weights of proteins. Therefore, it is not easy to display the full-length blots. However, having the original cropped films, we display the blots of repeated data with several exposure time (exposure 1 and 2) in the supplementary information following the editor’s suggestion.

Figure 1A.

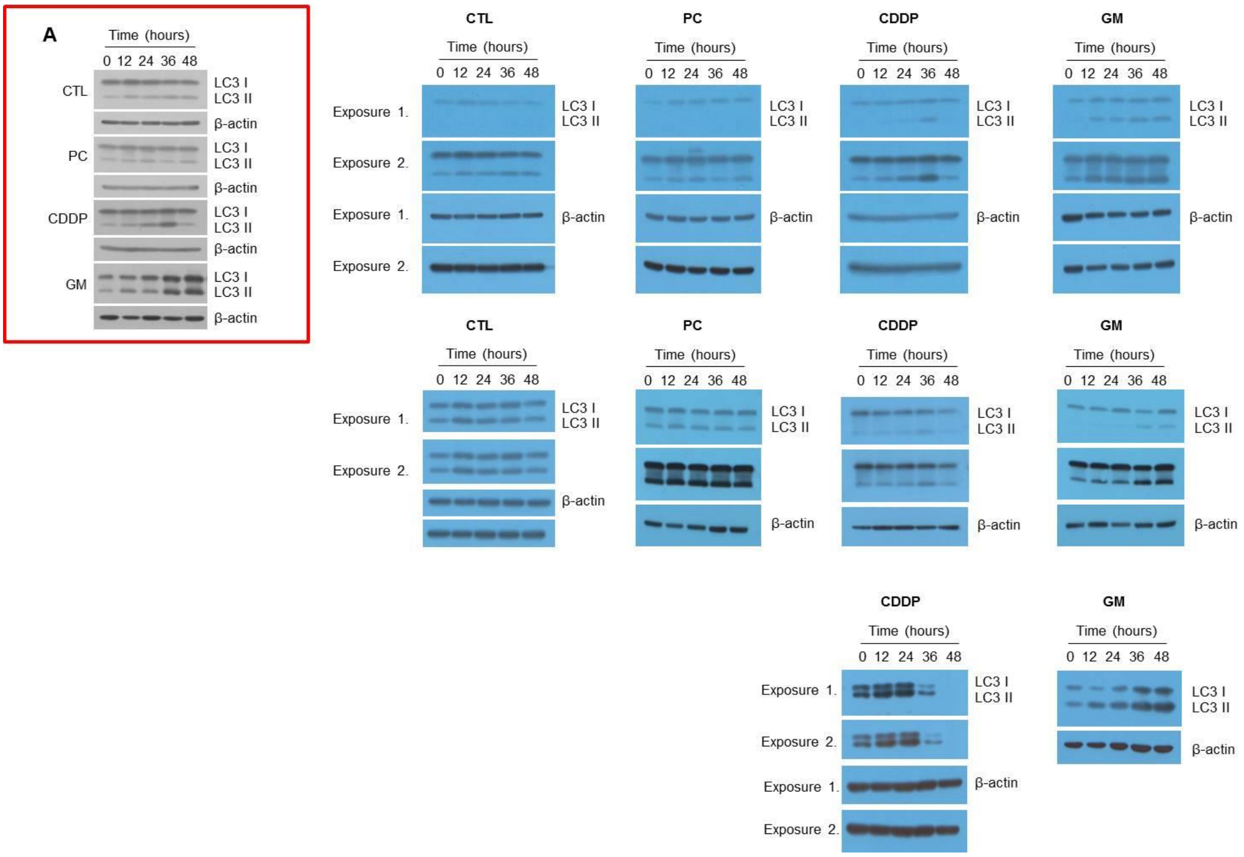

Figure 1F.

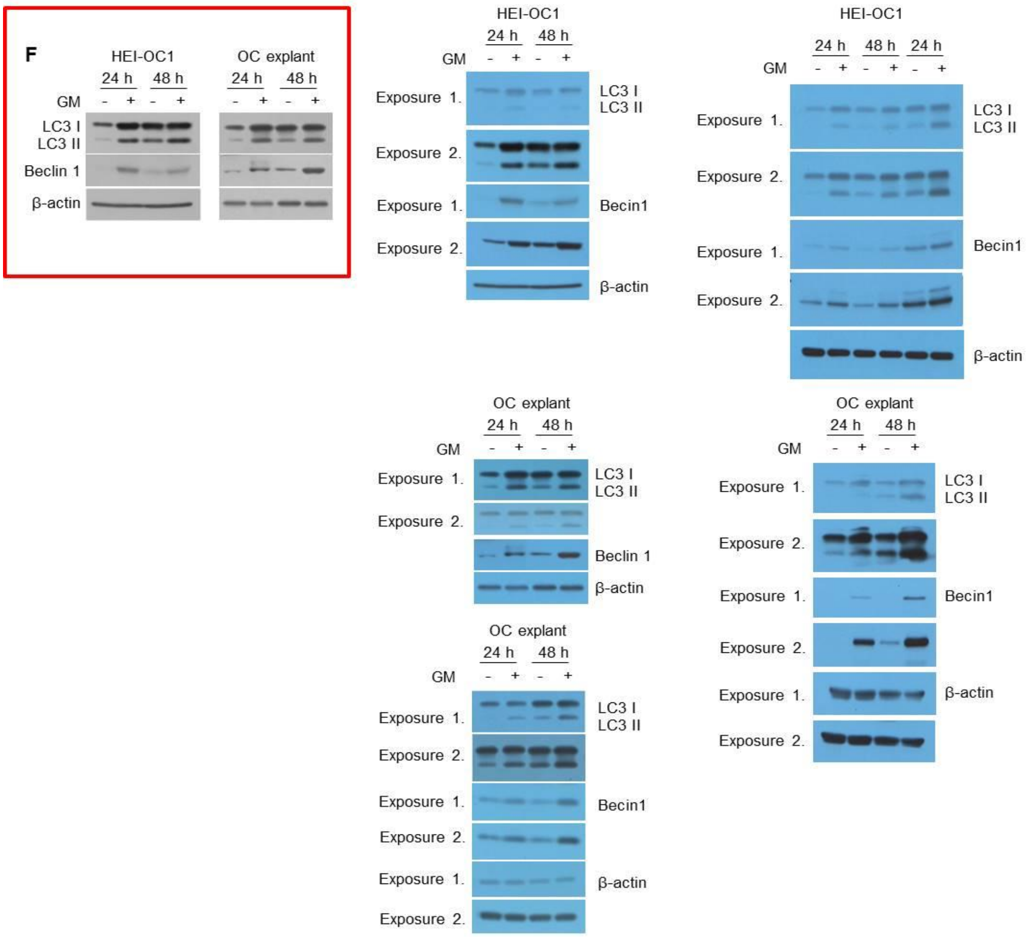

Figure 3A.

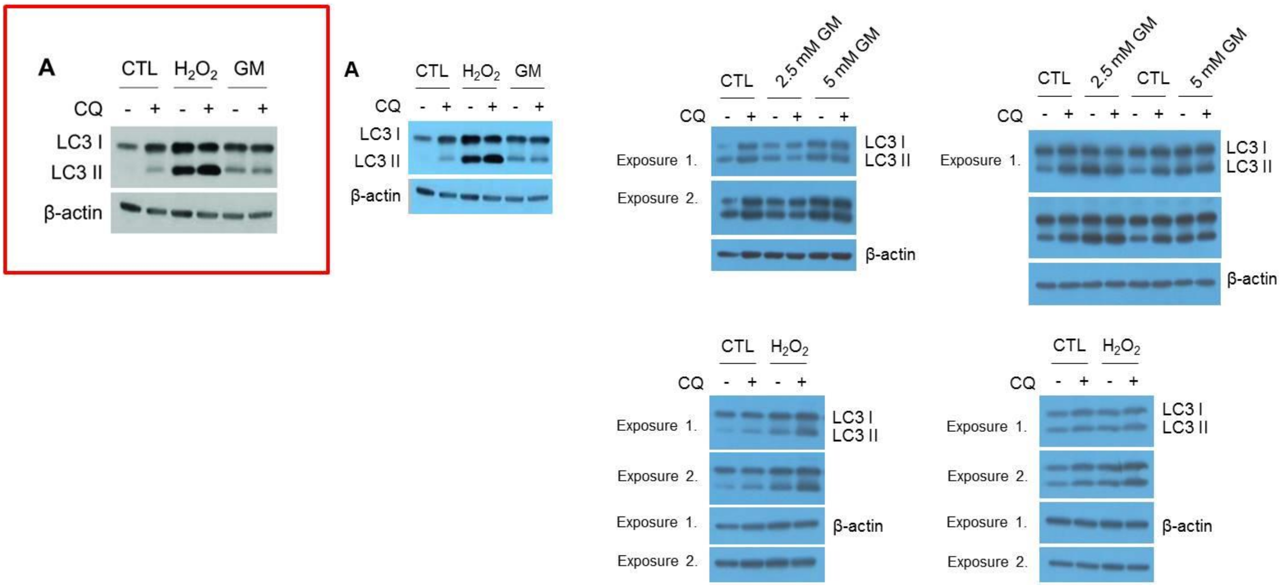

Figure 3B.

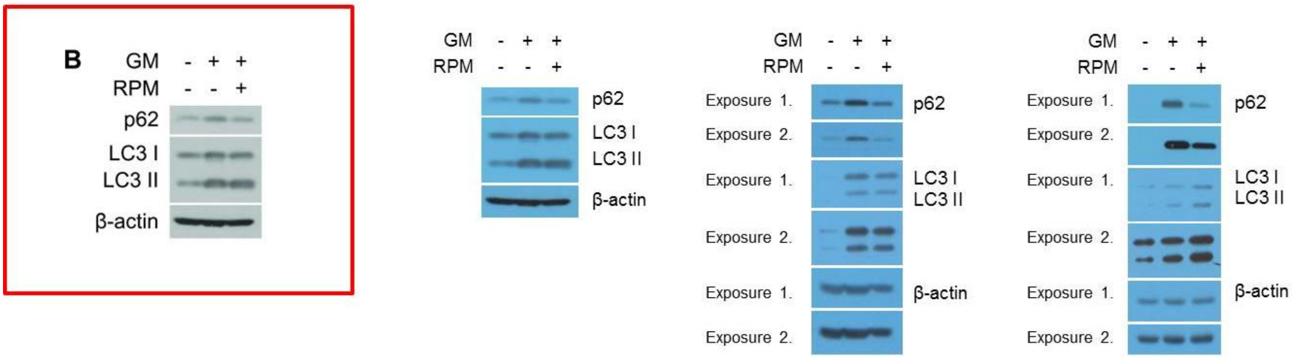

Figure 3E.

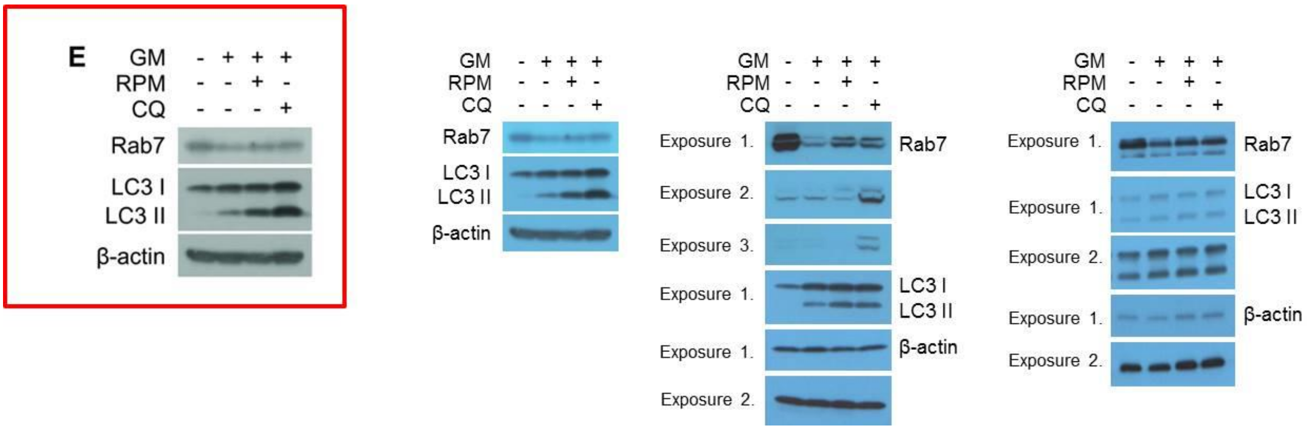

Supplement: Supplementary Information [file srep41356-s1.pdf]
